# A Novel Germline c.1267T>A *MEN1* Mutation in MEN1 Family—from Phenotype to Gene and Back

**DOI:** 10.3390/genes11111382

**Published:** 2020-11-21

**Authors:** Wojciech Gierlikowski, Agata Skwarek-Szewczyk, Michał Popow

**Affiliations:** Chair and Department of Internal Medicine and Endocrinology, Medical University of Warsaw, Banacha 1a, 02-097 Warsaw, Poland; agathaskwarek@yahoo.com (A.S.-S.); mpopow@wum.edu.pl (M.P.)

**Keywords:** MEN1, multiple endocrine neoplasia type 1, Menin, primary hyperparathyroidism, pituitary adenoma, neuroendocrine neoplasm, NGS, next generation sequencing, novel mutation

## Abstract

Primary hyperparathyroidism is a relatively common endocrine disorder, which may be hereditary. This report describes clinical, biochemical, radiographic, and genetic findings, the latter obtained using next generation sequencing (NGS), in three consanguineous patients. Gene panels in NGS consisted of 5 or 70 genes, including *MEN1* and *RET*. The first patient suffered from recurrent primary hyperparathyroidism. Primary hyperparathyroidism and pituitary microadenomas were afterwards diagnosed in two of her daughters. No clinical nor radiological features of gastroenteropancreatic neuroendocrine tumors were found. All three family members were heterozygous for *MEN1* NM_130799: c.1267T>A transversion, which is predicted to result in substitution of tryptophan with arginine in position 423. Additionally, the first patient was also a carrier of *RET* NM_020975: c.1946C>T missense mutation, which was not present in two other family members. We describe a family with a novel heterozygous mutation (NM_130799: c.1267T>A) in *MEN1* gene and postulate that it leads to MEN1 syndrome. The study underlies the importance of genetic testing in primary hyperparathyroidism in personalizing patients’ care.

## 1. Introduction

The estimated prevalence of primary hyperparathyroidism (PHPT) is about 2% and increases with a person’s age [1]. It is defined as elevated concentrations of calcium (total calcium corrected for albumin level or ionized calcium), elevated parathormone concentration (PTH; or normal, but not suppressed by hypercalcemia) and exclusion of secondary causes of hyperparathyroidism. Normocalcemic PHPT is a condition in which calcium concentration is normal, but PTH is elevated (and secondary causes of hyperparathyroidism are excluded) [2].

Approximately 10% of cases of PHPT are hereditary and may be classically divided into syndromic or non-syndromic disease (Table 1) [3]. Syndromic hereditary primary hyperparathyroidism occurs as a part of multiple endocrine neoplasia syndromes (MEN), occurring as type 1, 2, 4, and hyperparathyroidism-jaw tumor syndrome (HPT-JT). Non-syndromic congenital disease, also known as familial isolated hyperparathyroidism (FIHP), may also occur due to heterozygous germline mutations of the *MEN1* and *CDC73* (previously known as *HRPT2*) genes, but the mechanisms determining the altered phenotypic expressions of these mutations remain unknown [4,5,6]. Benign hypercalcemia with hypocalciuria may result from *CASR* (in familial hypocalciuric hypercalcemia, FHH), *GNA11* and *AP2S1* (in FHH-like cases) mutations [7].

Taking into account the reasons mentioned above, the American Association of Endocrine Surgeons parathyroidectomy guidelines [17] and The European Society of Endocrine Surgeons [18] recommend genetic testing in patients with PHPT who are younger than 40 years old, and who have family history of hypercalcemia or multi-gland disease. As each of the abovementioned genes may be affected by numerous mutations, sequencing of targeted gene panel employing next-generation sequencing (NGS) may be useful, however some factors, including revealing variants of unknown significance, may compromise the utility of the result [19,20,21].

Multiple endocrine neoplasia type 1 syndrome (MEN1) is an autosomal-dominant hereditary disorder that is characterized by a predisposition to, in declining percentage, tumors of the parathyroid glands, anterior pituitary, and neuroendocrine neoplasms (mainly pancreatic, (pNEN)). It is caused by mutation in *MEN1* gene, encoding for Menin. Menin is a 610-amio-acid protein, which locates in nucleus and acts as a tumor suppressor [22,23]. Clinically MEN1 is defined as the presence of two or more primary MEN1-related tumor types, or the presence of one of the MEN1-associated tumors in the first-degree relative of an MEN1 patient [8]. Primary hyperparathyroidism in MEN1 patients usually occurs by the age of 50, and is the initial manifestation of the disorder in most of them [24]. Conversely, up to 18% of patients with hyperparathyroidism may be affected by *MEN1* mutations [8]. Importantly, there are no strong genotype–phenotype correlations [5,25].

In this report we present a three-person family affected by MEN1 caused by the novel mutation, NM_130799: c.1267T>A, expected to lead to substitution of tryptophan with arginine in position 423.

## 2. Materials and Methods

Laboratory tests, imaging studies and pathological assessment were performed at the Medical Center of Medical University of Warsaw. Genetic testing was performed by Warsaw Genomics using NGS. Patient 1 was subjected to sequencing of a *CASR*, *CDC73*, *CDKN1B*, *MEN1* and *RET* multigene panel, with coverage of at least 75-fold and a quality score of at least 97.4% (for *RET*, 100% for other genes). Patients 2 and 3 were subjected to sequencing of 70 genes (listed in Table S1), among others *MEN1 RET*, and *CDKN2A*. The results were compared with multiple databases (1000GP, ClinVar, ConsesnusPathDB, Exome Aggregation Consortium, Exome Variant Server, FATHMM, Gene Ontology, Genotype-Tissue Expression, Genome Wide Association Study, HGMD, KEGG, MetaLR, MetaSVM, MutationAssessor, MutationTaster, OMIM, PolyPhen-2, PROVEAN, SIFT, SnpEff, dbNSFP, UniProt, Variant Effect Predictor). In case of detection of new mutation, the possibility of its pathogenicity was assessed in silico basing on expected amino-acids alterations. Additionally, we analyzed predicted protein alteration pathogenicity using FATHMM, PROVEAN, SIFT, PolyPhen-2, I-Mutant Suite, PANTHER, PhD-SNP, SNAP, Meta-SNP and PredictSNP tools.

All patients gave written consent for anonymous use of their medical data for scientific purposes.

## 3. Case Presentation

### 3.1. Patient 1

A 53-year-old female was referred to our Endocrinology Department due to suspicion of recurrent hyperparathyroidism. She had undergone open bilateral exploration with subtotal parathyroidectomy 8 years before. Notably, she reported that her father had also suffered primary hyperparathyroidism. The diagnosis of recurrent primary hyperparathyroidism was confirmed.

Due to clinical findings, the patient’s history, and family history, she was referred for genetic studies. *CASR*, *CDC73*, *CDKN1B*, *MEN1* and *RET* genes were sequenced, using an NGS panel, revealing two mutations: NM_020975: c.1946C>T and NM_130799: c.1267T>A in *RET* and *MEN1*, respectively. Both mutations were not found in the databases. Further assessment led to the conclusion, that due to predicted amino-acids substitutions (p.Ser649Leu and p.Trp423Arg, respectively), taking into account in silico analysis and the clinical picture, mutations should be classified as potentially pathogenic. Results of analysis of the altered Menin sequence can be found in Table S2.

The patient is now 55. Active surveillance for recurrent primary hyperparathyroidism is continued, with mildly elevated calcium and PTH concentrations. Diagnostics for renal calculi and osteoporosis was negative. She was also diagnosed with intraductal papillary mucinous neoplasm (IPMN) of the tail of pancreas, which was resected, and bilateral, non-functioning adrenals tumors (with radiographic features of adenomas). Importantly, endoscopic ultrasonography (EUS) did not reveal any other lesions. Prolactin concentration was not elevated.

### 3.2. Patient 2

**Figure 1:**
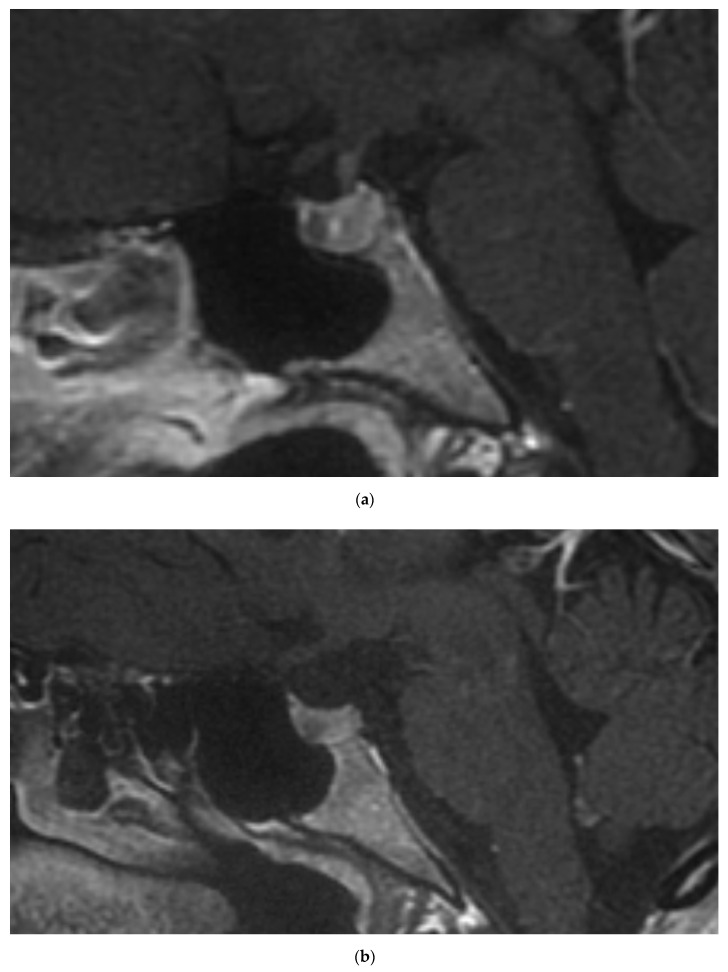
Contrast-enhanced magnetic resonance imaging revealed pituitary microadenomas in patient 2 (**a**) and patient 3 (**b**).

The 35-year-old daughter of the patient 1 is currently being observed due to mild PHPT. She was confirmed to inherit the *MEN1* c.1267T>A mutation, while no *RET* mutations were identified. Further assessment identified mild hyperprolactinemia, without menses disturbances and gonadotropins suppression. Pituitary gland magnetic resonance imaging (MRI) revealed presence of microadenoma (Figure 1a). Other imaging studies, including neck ultrasound and abdominal contrast-enhanced computed tomography (CT), did not revealed abnormalities.

### 3.3. Patient 3

The 33-year-old daughter of the patient 1 also inherited the *MEN1* c.1267T>A mutation, but not the *RET* mutation. She has been diagnosed with PHPT with enlargement of the right inferior parathyroid in neck ultrasound and hyperprolactinemia with 5mm lesion in pituitary with features of microadenoma on MRI (Figure 1b). Contrast-enhanced abdominal CT was normal.

Summary of findings is presented in Table 2.

## 4. Discussion

Primary hyperparathyroidism is a rare disease in young adults and its occurrence in these patients suggests the possibility of hereditary disease. Taking into account its longstanding asymptomatic course, it could be clinically presenting at any age, so that thorough family history is crucial and genetic testing is often needed [1]. *MEN1, RET*, *CASR*, and *CDC73* are thought to be mutated in more than a half of all cases of hyperparathyroidism with genetic background [26]. Because of recurrent disease and positive family history, patient 1 was recommended to undergo genetic studies, with an NGS panel containing five genes: *MEN1*, *RET*, *CASR*, *CDC73*, and *CDKN1B*. NGS revealed previously unknown mutations in *MEN1* and *RET*. Both mutations were missense ones and were reported as potentially pathogenic.

As a clinical picture and NGS results were intriguing, we assessed the proband’s first degree relatives—two daughters, who were in their fourth decade of life. Indeed, both had hypercalcemia and high PTH. Subsequently, we referred them for genetic testing and found out that they inherited *MEN1*, but not the *RET* mutation. We noted also mild hyperprolactinemia, however without clinical symptoms. Considering possible genetic background, we referred them to pituitary MRI, identifying lesions of features of microadenoma in both of them. A diagnosis of MEN1 may be established by fulfilling one of three criteria: the occurrence of two or more primary MEN1-associated endocrine tumors; the occurrence of one of the MEN1-associated tumors in a first-degree relative of a patient with a clinical diagnosis of MEN1; or identification of a germline *MEN1* mutation [8]. In our case, this pathway “from phenotype to gene and back” allow us to clinically diagnose MEN1 syndrome in Patients 2 and 3, and if so, also in Patient 1, and to postulate the pathogenicity of the identified *MEN1* mutation.

Our postulation regarding pathogenicity is supported by bioinformatics analysis and previous reports on the c.1267T>C mutation, which leads to the replacement of a tryptophan with arginine in position 423 (in ClinVar reported as NM_000244.3 (MEN1):c.1282T>C (p.Trp428Arg), accession VCV000457284.3, and interpreted as a variant of unknown significance). It was identified in a sporadic MEN1 case, in whom it manifested as PHPT and insulinoma [27]. Canaff et al. showed that c.1267T>C mutation leads to decreased Menin expression due to its rapid degradation [28]. A somatic c.1267T>C was also found in pNEN, according to the COSMIC database (legacy identifier COSM4135692), however the secretory function of the tumor is not mentioned [29]. These findings stand in line with our in silico analysis.

The mutation seems to result in multiple endocrine neoplasia type 1 syndrome with primary hyperparathyroidism and pituitary microadenoma but without neuroendocrine neoplasms in our observation. It stands in line with most common manifestations of MEN1 and is similar to a recently published report of our colleagues [30]. Nevertheless, we must emphasize, that there is no strict genotype–phenotype correlation in MEN1 [31,32], so all the patients need further screening for all MEN1-related tumors.

Genetic testing for germline mutations is very helpful in clinical practice, as some *RET* mutations require open neck exploration and early prophylactic thyroidectomy. Furthermore, an increased risk of parathyroid gland carcinoma and other malignancies is directly related to dysfunction of the *CDC73* gene and indicates careful removal of the tumor along with surrounding tissues [33]. Avoidance of minimally invasive parathyroid surgery, as well as arm transplantation of the parathyroid gland(s), and screening for associated tumors should be performed in patients with *MEN1* mutation. Gastroenteropancreatic NENs (GEP-NENs) are the component of MEN1 which leads to premature mortality, thus the possibility of its early diagnosis due to screening is the main benefit for identified MEN1 patients [34]. At the other end of the spectrum, benign hypercalcemia in familial hypocalciuric hypercalcemia (FHH), which is due to *CASR*/*GNA11*/*AP2S1* mutations, requires follow up, usually without the necessity of surgical treatment [7].

Phenotypes similar to MEN1 may be related to mutations in some Cyclin-Dependent Kinase Inhibitor Genes (*CDKIs*), namely *CDKN2B*, *CDKN2C*, *CDKN1A*, and *CDKN1B*, encoding for p15, p18, p21, and p27, respectively [10]. *CDKN2B*, *CDKN2C*, and *CDKN1A* were not sequenced, which is probably the most important limitation of the study. NGS data were not verified using Sanger sequencing, but such a verification is currently not considered as essential [35].

## 5. Conclusions

In conclusion, we postulate that the novel NM_130799: c.1267T>A mutation in *MEN1*, expected to result in p.Trp423Arg substitution, leads to MEN1. As most of the known *MEN1* variants are classified as being of unknown significance, this report on novel pathogenic *MEN1* mutation should be helpful for both healthcare providers and patients, leading to better care, especially for proper screening for GEP-NENs and thus reduction in premature mortality rate.

## Figures and Tables

**Table 1:** Hereditary primary hyperparathyroidism.

Syndrome	Gene	Hyperparathyroidism	Concomitant Diseases	Reference
MEN 1	*MEN*	Multiple adenoma	Neuroendocrine neoplasms (mainly pancreatic), pituitary adenoma	[8]
MEN 2	*RET*	Single adenoma	Phaeochromocytoma, thyroid medullary carcinoma	[9]
MEN 4	*CDKN1A* *CDKN1B* *CDKN2B* *CDKN2C*	Multiple adenoma	Neuroendocrine neoplasms (mainly pancreatic), pituitary adenoma, adrenal gland adenoma	[10]
FHH	*CASR*	None	FIHP	[11]
FHH-like phenotype	*GNA11* *AP2S1*	None	FIHP	[12]
HRPT2 (HPT-JT)	*CDC73*	Single adenoma	Parathyroid cancer, tumors of kidney, jejunum, uretero-urinary tract, and lungs	[13]
HRPT3	*PPP3R1* *GPR73*	Multiple adenoma	Neuroendocrine neoplasms, pituitary adenoma, adrenal gland adenoma	[14,15]
HRPT4	*GCM2*	Single adenoma	FIHP	[16]

MEN–multiple endocrine neoplasia syndrome; FHH—familial hypocalciuric hypercalcemia; FIHP—familial isolated hyperparathyroidism; HRPT–hyperparathyroidism; HPT-JT–hyperparathyroidism with jaw tumor.

**Table 2:** Abnormalities found in the patients.

	Patient 1	Patient 2	Patient 3
PHPT	yes	yes	yes
iPTH (15–65 (pg/mL))	69.7	61.2	79.40
Ca (2.15–2.60 (mmol/L))	2.72	2.62	2.71
Pi (0.81–1.45 (mmol/L))	1.07	0.75	0.78
Vitamin D3 total (ng/ mL)	32.05	33.61	33.3
Prolactin (4.79–23.3 ng/mL)	10.6	39.4	36.2
Pituitary MRI	no data	adenoma 4 × 3 × 4 mm	adenoma 4 × 5 × 3 mm
Abdominal imaging–pancreas	IPMN, 30 mm diameter (CT/EUS and histopathology)	4 mm cyst (CT)	no pathologies on CT
Abdominal imaging–adrenal glands	left: 16 × 11 mm, right: 11 × 7 mm and 9 × 7 mm, all densities <10HU (CT)	no pathologies on CT	no pathologies on CT
Genetic alterations	*MEN1* NM_130799: c.1267T>A *RET* NM_020975: c.1946C>T	*MEN1* NM_130799: c.1267T>A	*MEN1* NM_130799: c.1267T>A

PHPT—primary hyperparathyroidism, iPTH—intact parathyroid hormone, MRI—magnetic resonance imaging, IPMN—intraductal papillary mucinous neoplasm, CT—computed tomography, EUS—endoscopic ultrasonography, HU—Hounsfield unit.

## Supplementary Materials

The following are available online at https://www.mdpi.com/2073-4425/11/11/1382/s1, Table S1: List of genes sequenced in the case of patients 2 and 3. Table S2. Results of in silico analysis of pathogenicity of predicted protein sequence.

## References

[1] Falchetti A., Marini F., Giusti F., Cavalli T., Brandi M.L., Cavalli L. (2009). DNA-based test: When and why to apply it to primary hyperparathyroidism clinical phenotypes. J. Intern. Med..

[2] Dawood N.B., Yan K.L., Shieh A., Livhits M.J., Yeh M.W., Angela M. (2020). Leung Normocalcaemic primary hyperparathyroidism: An update on diagnostic and management challenges. Clin. Endocrinol..

[3] Carling T. (2001). Molecular pathology of parathyroid tumors. Trends Endocrinol. Metab..

[4] Dwight T., Twigg S., Delbridge L.W., Wong F., Farnebo F., Richardson A.L., Nelson A., Zedenius J., Philips J., Larsson C. (2000). Loss of heterozygosity in sporadic parathyroid tumours: Involvement of chromosome 1 and the MEN1 gene locus in 11q13. Clin. Endocrinol..

[5] Concolino P., Rossodivita A., Carrozza C., Raffaelli M., Lombardi C.P., Rigante D., Pitocco D., Stabile A., Bellantone R., Zuppi C. (2008). A novel MEN1 frameshift germline mutation in two Italian monozygotic twins. Clin. Chem. Lab. Med..

[6] Cardoso L., Stevenson M., Thakker R.V. (2017). Molecular genetics of syndromic and non-syndromic forms of parathyroid carcinoma. Hum. Mutat..

[7] Lee J.Y., Shoback D.M. (2018). Familial hypocalciuric hypercalcemia and related disorders. Best Pr. Res. Clin. Endocrinol. Metab..

[8] Thakker R.V., Newey P.J., Walls G.V., Bilezikian J., Dralle H., Ebeling P.R., Melmed S., Sakurai A., Tonelli F., Brandi M. (2012). Clinical Practice Guidelines for Multiple Endocrine Neoplasia Type 1 (MEN1). J. Clin. Endocrinol. Metab..

[9] Mulligan L.M., Ponder B.A. (1995). Genetic basis of endocrine disease: Multiple endocrine neoplasia type 2. J. Clin. Endocrinol. Metab..

[10] Agarwal S.K., Mateo C.M., Marx S.J. (2009). Rare Germline Mutations in Cyclin-Dependent Kinase Inhibitor Genes in Multiple Endocrine Neoplasia Type 1 and Related States. J. Clin. Endocrinol. Metab..

[11] Gorvin C.M., Frost M., Malinauskas T., Cranston T., Boon H., Siebold C., Jones E.Y., Hannan F.M., Thakker R.V. (2018). Calcium-sensing receptor residues with loss- and gain-of-function mutations are located in regions of conformational change and cause signalling bias. Hum. Mol. Genet..

[12] Hovden S., Rejnmark L., Ladefoged S.A., Nissen P.H. (2017). AP2S1 and GNA11 mutations—Not a common cause of familial hypocalciuric hypercalcemia. Eur. J. Endocrinol..

[13] Carpten J.D., Robbins C.M., Villablanca A., Forsberg L., Presciuttini S., Bailey-Wilson J., Simonds W.F., Gillanders E.M., Kennedy A.M., Chen J.D. (2002). HRPT2, encoding parafibromin, is mutated in hyperparathyroidism–jaw tumor syndrome. Nat. Genet..

[14] Lodefalk M., Frykholm C., Esbjörner E., Ljunggren Ö. (2015). Hypercalcaemia in a Patient with 2p13.2-p16.1 Duplication. Horm. Res. Paediatr..

[15] Warner J.V., Nyholt D.R., Busfield F., Epstein M., Burgess J., Stranks S., Hill P., Perry-Keene D., Learoyd D., Robinson B. (2005). Familial isolated hyperparathyroidism is linked to a 1.7 Mb region on chromosome 2p13.3-14. J. Med. Genet..

[16] Guan B., Welch J.M., Sapp J.C., Ling H., Li Y., Johnston J.J., Kebebew E., Biesecker L.G., Simonds W.F., Marx S.J. (2016). GCM2—Activating Mutations in Familial Isolated Hyperparathyroidism. Am. J. Hum. Genet..

[17] Wilhelm S.M., Wang T.S., Ruan D.T., Lee J.A., Asa S.L., Duh Q.-Y., Doherty G.M., Herrera M.F., Pasieka J.L., Perrier N.D. (2016). The American Association of Endocrine Surgeons Guidelines for Definitive Management of Primary Hyperparathyroidism. JAMA Surg..

[18] Bergenfelz A.O.J., Hellman P., Harrison B., Sitges-Serra A., Dralle H. (2009). Positional statement of the European Society of Endocrine Surgeons (ESES) on modern techniques in pHPT surgery. Langenbeck’s Arch. Surg..

[19] Selkirk C.G., Vogel K.J., Newlin A.C., Weissman S.M., Weiss S.M., Wang C.-H., Hulick P.J. (2014). Cancer genetic testing panels for inherited cancer susceptibility: The clinical experience of a large adult genetics practice. Fam. Cancer.

[20] Taylor J.C., Martin H.C., Lise S., Broxholme J., Cazier J.-B., Rimmer A., Kanapin A., Lunter G., Fiddy S., Allan C. (2015). Factors influencing success of clinical genome sequencing across a broad spectrum of disorders. Nat. Genet..

[21] Adams D.R., Eng C.M. (2018). Next-Generation Sequencing to Diagnose Suspected Genetic Disorders. N. Engl. J. Med..

[22] Guru S.C., Goldsmith P.K., Burns A.L., Marx S.J., Spiegel A.M., Collins F.S., Chandrasekharappa S.C. (1998). Menin, the product of the MEN1 gene, is a nuclear protein. Proc. Natl. Acad. Sci. USA.

[23] Larsson C., Skogseid B., Öberg K., Nakamura Y., Nordenskjöld M. (1988). Multiple endocrine neoplasia type 1 gene maps to chromosome 11 and is lost in insulinoma. Nat. Cell Biol..

[24] Trump D., Farren B., Wooding C., Pang J.T., Besser G.M., Buchanan K.D., Edwards C.R., Heath D.A., Jackson C.E., Jansen S. (1996). Clinical studies of multiple endocrine neoplasia type 1 (MEN1). QJM.

[25] Marini F., Giusti F., Fossi C., Cioppi F., Cianferotti L., Masi L., Boaretto F., Zovato S., Cetani F., Colao A. (2018). Multiple endocrine neoplasia type 1: Analysis of germline MEN1 mutations in the Italian multicenter MEN1 patient database. Endocrine.

[26] Marx S.J., Goltzman D. (2019). Evolution of Our Understanding of the Hyperparathyroid Syndromes: A Historical Perspective. J. Bone Miner. Res..

[27] Cebrian A. (2003). Mutational and gross deletion study of the MEN1 gene and correlation with clinical features in Spanish patients. J. Med. Genet..

[28] Canaff L., Vanbellinghen J.-F., Kanazawa I., Kwak H., Garfield N., Vautour L., Hendy G.N. (2012). Menin Missense Mutants Encoded by the MEN1 Gene that Are Targeted to the Proteasome: Restoration of Expression and Activity by CHIP siRNA. J. Clin. Endocrinol. Metab..

[29] Tate J.G., Bamford S., Jubb H.C., Sondka Z., Beare D.M., Bindal N., Boutselakis H., Cole C.G., Creatore C., Dawson E. (2019). COSMIC: The Catalogue of Somatic Mutations in Cancer. Nucleic Acids Res..

[30] Stasiak M., Dedecjus M., Zawadzka-Starczewska K., Adamska E., Tomaszewska M., Lewiński A. (2020). Novel Germline c.105_107dupGCT MEN1 Mutation in a Family with Newly Diagnosed Multiple Endocrine Neoplasia Type 1. Genes.

[31] Soczomski P., Jurecka-Lubieniecka B., Rogozik N., Tukiendorf A., Jarząb B., Bednarczuk T. (2019). Multiple endocrine neoplasia type 1 in Poland: A two-centre experience. Endokrynol. Polska.

[32] Lemos M.C., Thakker R. (2008). Multiple endocrine neoplasia type 1 (MEN1): Analysis of 1336 mutations reported in the first decade following identification of the gene. Hum. Mutat..

[33] Shattuck T.M., Välimäki S., Obara T., Gaz R.D., Clark O.H., Shoback D., Wierman M.E., Tojo K., Robbins C.M., Carpten J.D. (2003). Somatic and Germ-Line Mutations of theHRPT2Gene in Sporadic Parathyroid Carcinoma. N. Engl. J. Med..

[34] Kamilaris C.D.C., Stratakis C.A. (2019). Multiple Endocrine Neoplasia Type 1 (MEN1): An Update and the Significance of Early Genetic and Clinical Diagnosis. Front. Endocrinol..

[35] Beck T.F., Mullikin J.C., Biesecker L.G., Program T.N.C.S. (2016). Systematic Evaluation of Sanger Validation of Next-Generation Sequencing Variants. Clin. Chem..

