# Supplementary material for: A Novel Germline c.1267T>A MEN1 Mutation in MEN1 Family—from Phenotype to Gene and Back
Source: Genes (Basel). 2020 Nov 21;11(11):1382. doi: 10.3390/genes11111382 (PMC7700542; doi:10.3390/genes11111382)
Supplement: Supplementary file 1 [file genes-11-01382-s001.pdf]

Table S1. List of genes sequenced in the case of patients 2 and 3.

AKT1; APC; ATM; ATP9B, AXIN2; BARD1; BMPR1A; BRCA1; BRCA2; BRIP1; CDH1; CDKN2A; CHEK2; CTNNA1; CYP21A2; DIRC3; EPCAM; EXO1; FANCC; FH; FLCN; GALNT12; GDNF; GREM1; HNF1A; HNF1B; KIF1B; MAX; MC1R; MEN1; MET; MITF; MLH1; MLH3; MRE11; MSH2; MSH6; MUTYH; NBN; NF1; PALB2; PIK3CA; PMS1; PMS2; POLD1; POLE; POT1; PRKAR1A; PRSS1; PTCH1; PTEN; RAD51C; RAD51D; RET; SDHA; SDHAF2; SDHB; SDHC; SDHD; SMAD4; STK11; TGFBR2; TMEM127; TP53; TSC1; TSC2; VHL; WT1; XRCC2; XRCC3

Table S2. Results of in silico analysis of pathogenicity of predicted protein sequence.

**Algorithm**	**Score**	**Interpretation**
FATHMM	0.99	pathogenic
PROVEAN	-12.393	deleterious
SIFT	0.00	disease
PolyPhen-2	1.00	probably damaging
I-Mutant Suite	(RI) 7	disease
PANTHER	0.826	disease
PhD-SNP	0.871	disease
SNAP	0.780	disease
Meta-SNP	0.785	disease
PredictSNP	87%	deleterious
